# The effect of combining nutrient intake and physical activity levels on central obesity, sarcopenia, and sarcopenic obesity: a population-based cross-sectional study in South Korea

**DOI:** 10.1186/s12877-023-03748-x

**Published:** 2023-03-03

**Authors:** Jong Eun Park, Seulgi Lee, Kirang Kim

**Affiliations:** 1 Institute of Health & Science Convergence, Chungbuk National University, 28644 Cheongju, South Korea; 2 Department of Food Science and Nutrition, Dankook University, 119 Dandae-ro, Dongnam-gu, 31116 Cheonan, South Korea

**Keywords:** Sarcopenia, Sarcopenic obesity, Central obesity, Energy, Protein, Physical activity

## Abstract

**Background:**

This study was conducted to investigate the effects of combining nutritional and physical activity (PA) factors on four different categories, according to the presence or absence of sarcopenia and central obesity.

**Methods:**

From the 2008–2011 Korea National Health and Nutrition Examination Survey, 2971 older adults aged ≥ 65 years were included and divided into four groups based on their sarcopenia and central obesity status: healthy control (39.3%), central obesity (28.9%), sarcopenia (27.4%), and sarcopenic obesity (4.4%). Central obesity was defined as a waist circumference of ≥ 90 cm in men and ≥ 85 cm in women. Sarcopenia was defined as an appendicular skeletal mass index of < 7.0 kg/m^2^ in men and < 5.4 kg/m^2^ in women, and sarcopenic obesity was defined as the coexistence of sarcopenia and central obesity.

**Results:**

Participants who consumed more energy and protein than the average requirement had a lower likelihood of having sarcopenia (odds ratio (OR): 0.601, 95% confidence interval (CI): 0.444–0.814) than those who did not consume enough nutrients. The likelihood of central obesity and sarcopenic obesity decreased in groups with recommended PA levels, regardless of whether energy intake met or did not meet the average requirement. Whether PA met or did not meet the recommended level, the likelihood of sarcopenia decreased in groups with energy intake that met the average requirement. However, when PA and energy requirements were met, there was a greater reduction in the likelihood of sarcopenia (OR: 0.436, 95% CI: 0.290–0.655).

**Conclusion:**

These findings suggest that adequate energy intake that meets requirements is more likely to be effective as a major prevention and treatment goal for sarcopenia, whereas PA guidelines should be prioritized in the case of sarcopenic obesity.

**Supplementary Information:**

The online version contains supplementary material available at 10.1186/s12877-023-03748-x.

## Background

Changes in body composition, including an increase in adipose tissue mass and a decrease in skeletal muscle mass, have age-related health implications for the older adults [1, 2]. A recent Korean meta-analysis for the prevalence of sarcopenia in the older adults found that it was 13.1% in the overall older adults, 14.9% in men, and 11.4% in women [3]. Obesity prevalence in older adults aged ≥ 70 years was 31.9% in men and 37.8% in women, according to the Korea National Health and Nutrition Examination Survey (KNHANES) [4]. Prevalence of central obesity based on waist circumference was 48.4% and 57.5% for men and women aged ≥ 70 years, respectively [5]. The double burden of these outcomes with aging is a major concern among the older adults.

Sarcopenia is characterized as “age-related loss of skeletal muscle mass plus loss of muscle strength and/or decreased physical performance” [6], and obesity is commonly defined as an excessive amount of body fat [7]. Sarcopenia and obesity, especially visceral obesity, affect each other via common pathophysiological mechanisms, such as changes in hormone levels, vascular changes, low-grade inflammation, and immunological factors [8]. In addition, sarcopenia is related to low physical activity (PA) and energy expenditure, which increases the risk of obesity, whereas obesity increases inflammation associated with the development of sarcopenia [9–11]. Thus, sarcopenia often coexists with obesity, and these comorbidities are often clinically observed in the older adults; The coexistence of low skeletal muscle mass and high adiposity levels, known as sarcopenic obesity, is a new category of obesity in older adults [7, 12]. Several previous studies have demonstrated that sarcopenic obesity may have a greater impact on worse cardiovascular risk profiles than either sarcopenia or obesity alone [7, 13, 14].

Sarcopenia and obesity are both modifiable health outcomes that can be managed by modifying risk factors [15, 16]. Although the risks of sarcopenia and obesity are multifactorial, they may be linked to lower rates of energy expenditure and inadequate dietary intake as age increases [17]. Thus, effective nutritional and exercise strategies are required to treat sarcopenia, obesity, or sarcopenic obesity. Previous research focused on combined nutrition and exercise strategies, including exercise with a hypocaloric diet or protein intake. However, despite being effective in reducing body weight, these interventions may result in skeletal muscle mass loss [15].

There are numerous effective strategies for simultaneously reducing body fat and increasing skeletal muscle mass, but evidence in older Asians with different dietary patterns and body sizes compared with Western older adults is limited. Korean older adults, in particular, have lower PA and energy intakes, with a high percentage of energy derived from carbohydrates [4]. Thus, optimal strategies for the target population must be developed to prevent and manage obesity, sarcopenia, and sarcopenic obesity. Therefore, this study aimed to investigate the combined effect of energy intake with macronutrients and exercise on obesity, sarcopenia, and sarcopenic obesity using the KNHANES dataset.

## Methods

### Data source and study population

This cross-sectional study used data from the Korea Disease Control and Prevention Agency (KDCA) 4th (2008–2009) and 5th (2010–2011) KNHANES. A complex, stratified, multistage probability cluster survey was used to select a representative sample of the South Korean non-institutionalized civilian population. The survey was designed to assess the Korean population’s health and nutritional status through health interviews, physical examinations, and nutrition surveys as previously described [18].

Of the 37,753 people who participated in the 4th and 5th KNHANES, a total of 6370 older adults aged ≥ 65 years were enrolled for this study. Subsequently, we excluded all participants who met the following exclusion criteria: (i) missing appendicular skeletal muscle mass data (n = 2205), (ii) missing height or waist circumference values (n = 25), (iii) non-participation in 24-h dietary recall interview or extreme energy intake (< 500 or > 5000 kcal/day) (n = 327), (iv) strict diet to manage disease or weight (n = 708), (v) failure to answer PA questionnaires (n = 49), and (vi) patients with severe diseases such as cancer, renal failure, and liver cirrhosis (n = 85). Finally, 3399 participants were excluded, leaving 2971 participants (1275 males and 1696 females) eligible for the final analysis (Supplementary Fig. 1).

### Definition of sarcopenia and central obesity

Muscle mass was measured using dual-energy X-ray absorptiometry (DXA) (Discovery-W fan-beam densitometer, Hologic, Inc., MA, USA), and appendicular skeletal muscle mass (ASM) was calculated by adding the lean muscle mass of both arms and legs. The appendicular skeletal muscle mass index (ASMI), which is used to diagnose sarcopenia, was calculated by dividing the ASM by the square of the height (kg/m^2^). Sarcopenia was defined as an ASMI of < 5.4 kg/m^2^ in women and < 7.0 kg/m^2^ in men [6]. The Asian Working Group for Sarcopenia (AWGS) proposed this cutoff as part of a diagnostic algorithm for sarcopenia in older Asians in light of ethnic differences in skeletal muscle mass.

Waist circumference was measured to the nearest 0.1 cm at the midpoint between the lower rib cage margin and the top of the iliac crest. According to the Korean Society for the Study of Obesity, central obesity was defined as a waist circumference of ≥ 90 cm in men and ≥ 85 cm in women [19]. Sarcopenic obesity was defined as the coexistence of both low ASM and central obesity.

All participants were divided into four groups based on their sarcopenia and central obesity status: healthy control (non-sarcopenic and non-central obese), central obesity (non-sarcopenic and central obese), sarcopenia (sarcopenic and non-central obese), and sarcopenic obesity (sarcopenic and central obese).

### Nutritional assessment

Dietary intake was assessed using a 24-h dietary recall method. Detailed information about all foods and beverages consumed by the participant in the previous 24 h was collected by trained dietitians. To compare the nutritional status of participants, the total daily energy and macronutrient intake, as well as the percentage of energy delivered by macronutrients, were calculated. The percentage of subjects consuming insufficient amounts of major nutrients was determined to be less than the estimated energy requirement (EER) or the estimated average requirement (EAR) using the revised 2020 Korean Dietary Reference Intakes (KDRIs) [20].

### PA assessment

PA was determined using a questionnaire about the frequency of moderate- or vigorous-intensity activities per week (how many times a week and how many minutes and hours at a time). The participants were divided based on their level of moderate- or vigorous-intensity PA per week into three groups: inactive (no PA), insufficient PA level, and recommended PA level. The “Recommended PA level” was defined as moderate-intensity PA for at least 150 min per week, or vigorous-intensity PA for at least 75 min per week, or a combination of moderate- and vigorous-intensity PAs for at least 75 min per week. When moderate- and vigorous-intensity PAs were combined, 1 min of vigorous-intensity PA was considered to correspond to 2 min of moderate-intensity PA [21].

### Other variables

Sociodemographic characteristics, including age (65 − 69, 70 − 74, 75 − 79, or ≥ 80 years), sex, marital status (spouse/partner present or absent), educational level (elementary school graduate or less, middle school graduate, high school graduate, or college graduate or more), and household income (first, second, third, and fourth quartile in each period), and health behavior variables, including smoking (never, former, or current smokers) and frequency of binge drinking in the previous year, and diabetes as a comorbid disease, were identified. Binge drinking was defined as consuming seven or more alcoholic drinks on a single occasion for men and five or more alcoholic drinks on a single occasion for women. Participants were divided into four groups based on their reported number of binge drinking episodes in the previous year: lifetime abstainer, never in the previous year, once a month or less, once a week, and almost daily. Diabetes was defined as a fasting plasma glucose of ≥ 126 mg/dL, or the use of oral hypoglycemic medications or insulin. Blood pressure and biochemical indicators such as triglycerides, high-density lipoprotein (HDL) cholesterol, and others, as well as body mass index (BMI) and body fat percentage were assessed directly as part of the physical examination using standardized techniques and methods. The components of the metabolic syndrome were defined as follows: high triglycerides (≥ 150 mg/ dL or drug treatment for lipid abnormality), low HDL cholesterol (< 40 mg/dL in men and < 50 mg/dL in women or drug treatment for lipid abnormality), high blood pressure (≥ 130/85 mm Hg or drug treatment for elevated blood pressure), and high fasting glucose (≥ 100 mg/dL or antidiabetic drug treatment) [22]. Body composition, including fat-free mass and body fat percentage, was measured by DXA scans.

### Statistical analysis

In all analyses, survey sample weights were used to generate estimates that were representative of the Korean population. The characteristics of groups based on sarcopenia and central obesity status were compared using the PROC SURVEYREG procedure for continuous variables and the PROC SURVEYFREQ procedure for categorical variables. Multinomial logistic regression was used to assess the relationship between nutrition and PA factors and sarcopenia and central obesity status, with odds ratios (ORs) and 95% confidence intervals (CIs) calculated for central obesity, sarcopenia, and sarcopenic obesity. Age, sex, marital status, educational level, household income, smoking, frequency of binge drinking, PA level, and diabetes were all included in the model as covariates. Statistical analyses were performed using the SAS statistical software version 9.4 (SAS Institute Inc., Cary, NC, USA). A *p*-value of < 0.05 was considered statistically significant.

## Results

The characteristics of the study subjects are shown in Table 1. A total of 2971 participants were included, with the healthy control, central obesity, sarcopenia, and sarcopenic obesity groups accounting for 39.3%, 28.9%, 27.4%, and 4.4%, respectively. The healthy control group had the lowest mean age (71.9 ± 0.2 years), whereas the sarcopenic obesity group had the highest (74.0 ± 0.5 years) (*p* < 0.001). Compared with the healthy control group (37.0%), central obesity (31.3%), sarcopenic obesity (42.9%), and sarcopenia groups (60.1%) had a higher frequency of men (*p* < 0.001). The proportion of married people was highest in the sarcopenia group (*p* = 0.003), with no significant difference between groups in socioeconomic characteristics such as educational level and household income. The proportion of current smokers was significantly higher in the sarcopenia group (41.5%) (*p* < 0.001), and the proportion of diabetes cases was significantly higher in the sarcopenic obesity group (24.2%) (*p* < 0.001).

**Table 1:** General characteristics of participants based on sarcopenia and central obesity status

	**Healthy control**	**Central obesity**	***p* value**	**Sarcopenia**	**Sarcopenic obesity**	***p* value**	***p* value**^**a**^
All subjects	1167	858		814	132		
Age, years (Mean ± SE)	71.9 ± 0.2	72.2 ± 0.2	0.213	73.5 ± 0.2	74.0 ± 0.5	0.337	< 0.001
65 − 69	456 (38.4)	345 (34.9)	0.503	210 (26.9)	28 (20.6)	0.658	< 0.001
70 − 74	375 (30.5)	295 (33.8)		251 (27.5)	46 (30.2)		
75 − 79	219 (19.6)	141 (20.2)		214 (26.8)	36 (28.3)		
≥ 80	117 (11.4)	77 (11.1)		139 (18.8)	22 (21.0)		
Sex			0.028			0.002	< 0.001
Male	432 (37.0)	269 (31.3)		512 (60.1)	62 (42.9)		
Female	735 (63.0)	589 (68.7)		302 (39.9)	70 (57.1)		
Marital status			0.288			0.017	0.003
Spouse/partner present	759 (61.7)	537 (58.7)		589 (68.9)	81 (56.0)		
Spouse/partner absent (separated, divorced, widowed, or never married)	406 (38.3)	318 (41.3)		222 (31.1)	51 (44.0)		
Educational level			0.943			0.092	0.290
Elementary school graduate or less	878 (75.0)	640 (75.1)		561 (69.9)	99 (79.0)		
Middle school graduate	113 (10.0)	93 (10.5)		106 (13.4)	18 (12.0)		
High school graduate	116 (9.8)	85 (9.9)		93 (10.3)	11 (6.8)		
College graduate or more	55 (5.2)	38 (4.5)		48 (6.4)	4 (2.1)		
Household income			0.386			0.172	0.125
First quartile (lowest)	618 (51.2)	445 (51.3)		471 (58.0)	65 (47.3)		
Second quartile	287 (25.1)	203 (23.3)		181 (23.1)	37 (28.4)		
Third quartile	148 (13.9)	98 (12.6)		78 (10.1)	22 (17.7)		
Fourth quartile (highest)	96 (9.8)	101 (12.8)		67 (8.9)	5 (6.6)		
Smoking			0.070			0.009	< 0.001
Never-smokers	739 (62.1)	581 (67.0)		340 (41.5)	72 (56.9)		
Former smokers	120 (11.7)	86 (11.8)		137 (17.0)	13 (10.1)		
Current smokers	307 (26.2)	191 (21.2)		337 (41.5)	47 (33.0)		
Frequency of binge drinking			0.971			0.301	0.602
Lifetime abstainer	611 (52.2)	443 (52.2)		375 (46.9)	78 (58.3)		
Never in the previous year	317 (26.9)	225 (25.6)		252 (30.4)	24 (21.0)		
Once a month or less	139 (12.1)	112 (12.7)		104 (11.4)	17 (10.9)		
Once a week	53 (5.2)	46 (5.8)		40 (6.0)	7 (5.2)		
Almost daily	46 (3.7)	31 (3.7)		41 (5.3)	6 (4.6)		
Presence of diabetes			< 0.001			0.019	< 0.001
No	981 (82.0)	614 (70.5)		612 (72.9)	89 (65.8)		
Yes	105 (9.3)	170 (18.5)		98 (13.2)	29 (24.2)		
Unknown	81 (8.7)	74 (11.0)		104 (13.9)	14 (10.0)		

Abbreviation: *KNHANES* Korea National Health and Nutrition Examination Survey

All data analyses conducted in the present study were based on weighted estimates with sample weight provided by KNHANES

Continuous variables are presented as mean ± standard error, and categorical variables are presented as n (%)

^a^ Indicates statistical significance between the four groups

All obesity indicators, including waist circumference, BMI, and total body fat percentage adjusted for age and sex, were significantly higher in the central obesity and sarcopenic obesity groups than in the healthy control and sarcopenia groups (Table 2; all *p* < 0.001). ASM and total fat-free mass were lower in the sarcopenic group than in the non-sarcopenic group, with the sarcopenic group having the lowest mean values (all *p* < 0.001). In terms of metabolic characteristics, triglycerides, blood pressure, and fasting glucose levels were significantly higher in the obese groups than in the healthy control and sarcopenia groups, while HDL-cholesterol levels were significantly lower (all *p* < 0.001).

**Table 2:** Anthropometric and metabolic characteristics of participants based on sarcopenia and central obesity status

	**Healthy control**	**Central obesity**	***p* value**	**Sarcopenia**	**Sarcopenic obesity**	***p* value**	***p* value**^**a**^
ASM (kg)^b^	16.7 ± 0.1	17.8 ± 0.1	< 0.001	14.1 ± 0.1	14.7 ± 0.1	0.005	< 0.001
ASM/height^2^ (kg/m^2^)^b^	6.74 ± 0.02	7.05 ± 0.03	< 0.001	5.71 ± 0.02	5.80 ± 0.04	0.084	< 0.001
Total fat-free mass (kg)^b^	41.4 ± 0.1	45.0 ± 0.2	< 0.001	36.7 ± 0.2	39.5 ± 0.3	< 0.001	< 0.001
Waist circumference (cm)^b^	79.9 ± 0.2	93.4 ± 0.2	< 0.001	76.2 ± 0.3	91.8 ± 0.4	< 0.001	< 0.001
BMI (kg/m^2^)^b^	22.8 ± 0.1	26.5 ± 0.1	< 0.001	20.7 ± 0.1	24.1 ± 0.2	< 0.001	< 0.001
Total body fat percentage (%)^b^	25.4 ± 0.2	31.5 ± 0.2	< 0.001	27.1 ± 0.3	34.2 ± 0.4	< 0.001	< 0.001
Metabolic syndrome components							
High triglycerides			< 0.001			0.004	< 0.001
No	705 (63.2)	398 (50.2)		481 (64.6)	54 (46.0)		
Yes	380 (36.8)	382 (49.8)		232 (35.4)	59 (54.0)		
Low HDL cholesterol			< 0.001			< 0.001	< 0.001
No	473 (42.6)	232 (29.7)		397 (55.2)	38 (32.0)		
Yes	612 (57.4)	548 (70.3)		316 (44.8)	75 (68.0)		
High blood pressure			< 0.001			0.034	< 0.001
No	415 (35.2)	192 (22.0)		285 (32.5)	28 (21.5)		
Yes	752 (64.8)	666 (78.0)		528 (67.5)	104 (78.5)		
High fasting glucose			< 0.001			0.013	< 0.001
No	737 (65.8)	374 (47.5)		449 (61.3)	52 (47.4)		
Yes	349 (34.2)	410 (52.5)		261 (38.7)	66 (52.6)		
Number of metabolic syndrome components present			< 0.001			< 0.001	< 0.001
0	118 (9.7)	34 (4.0)		89 (10.5)	2 (4.3)		
1	326 (29.6)	110 (13.6)		218 (30.8)	19 (12.9)		
2	306 (29.1)	232 (30.5)		221 (29.8)	35 (29.1)		
3	236 (22.4)	243 (32.1)		113 (18.5)	34 (36.7)		
4	91 (9.2)	156 (20.0)		65 (10.3)	22 (16.9)		

Abbreviation: *ASM* appendicular skeletal muscle mass, *BMI* body mass index, *HDL* high-density lipoprotein

All data analyses conducted in the present study were based on weighted estimates with sample weight provided by KNHANES

Continuous variables are presented as mean ± standard error, and categorical variables are presented as n (%)

^a^ Indicates statistical significance between the four groups

^b^ Adjusted for age and sex

After adjusting for age and sex, the total daily energy intake and the percentage of energy intake to EER were significantly lower in the sarcopenia group than in the non-sarcopenia group (Table 3; all *p* < 0.001). Both absolute fat intake (*p* = 0.036) and the percentage of energy from fat (*p* = 0.015) were the highest in the central obesity group. The proportion of individuals with a protein intake below the EAR was significantly lower in the sarcopenia group than in the non-sarcopenia group (*p* = 0.002). The proportion of individuals who met their energy and protein intake requirements was significantly higher in the non-sarcopenia group, but the proportion of those who did not was higher in the sarcopenia groups, particularly in the sarcopenic obesity group (54.3%) (*p* < 0.001).

**Table 3:** Daily energy and nutrient intake of participants based on sarcopenia and central obesity status

	**Healthy control**	**Central obesity**	***p* value**	**Sarcopenia**	**Sarcopenic obesity**	***p* value**	***p* value**^**a**^
Total daily intake							
Energy (kcal/day)^b^	1676.8 ± 22.6	1669.9 ± 29.8	0.860	1525.3 ± 25.3	1557.7 ± 47.7	0.587	< 0.001
Carbohydrate (g/day)^c^	301.0 ± 2.1	295.7 ± 2.7	0.087	294.5 ± 2.9	292.5 ± 5.4	0.783	0.138
Protein (g/day)^c^	52.0 ± 0.6	53.0 ± 0.7	0.208	52.2 ± 0.8	51.1 ± 1.4	0.460	0.500
Fat (g/day)^c^	20.5 ± 0.6	22.8 ± 0.7	0.004	21.3 ± 0.6	20.9 ± 1.2	0.685	0.036
Percentage of energy from macronutrients							
Percentage energy from carbohydrate (%)^c^	76.4 ± 0.3	75.0 ± 0.5	0.004	75.5 ± 0.4	75.7 ± 0.9	0.888	0.025
Percentage energy from protein (%)^c^	12.8 ± 0.1	13.0 ± 0.2	0.240	13.0 ± 0.1	12.8 ± 0.3	0.577	0.516
Percentage energy from fat (%)^c^	10.8 ± 0.3	12.0 ± 0.4	0.002	11.5 ± 0.2	11.5 ± 0.7	0.939	0.015
Intake of key nutrients compared to KDRIs							
Percentage of energy intake to EER (%)^b^	95.2 ± 1.3	95.2 ± 1.8	0.965	86.8 ± 1.4	88.6 ± 2.8	0.540	< 0.001
Level of energy intake			0.454			0.568	< 0.001
< EER	695 (59.1)	522 (61.3)		574 (70.7)	94 (67.9)		
≥ EER	472 (40.9)	336 (38.7)		240 (29.3)	38 (32.1)		
Percentage of protein intake to RNI (%)^c^	93.6 ± 1.0	96.0 ± 1.3	0.097	94.1 ± 1.4	92.0 ± 2.6	0.519	0.307
Level of protein intake			0.747			0.160	0.002
< EAR	499 (42.8)	338 (41.8)		407 (50.3)	77 (57.9)		
≥ EAR	668 (57.2)	520 (58.2)		407 (49.7)	55 (42.1)		
Different levels of energy and protein intake			0.441			0.201	< 0.001
Energy intake ≥ EER, protein intake ≥ EAR	438 (38.1)	320 (37.1)		212 (25.9)	35 (28.5)		
Energy intake ≥ EER, protein intake < EAR	34 (2.8)	16 (1.6)		28 (3.4)	3 (3.6)		
Energy intake < EER, protein intake ≥ EAR	230 (19.1)	200 (21.1)		195 (23.8)	20 (13.6)		
Energy intake < EER, protein intake < EAR	465 (40.0)	322 (40.2)		379 (46.9)	74 (54.3)		

Abbreviation: KDRIs, Korean dietary reference intakes; EER, estimated energy requirement; RNI, recommended nutrient intake; EAR, estimated average requirement

All data analyses conducted in the present study were based on weighted estimates with sample weight provided by KNHANES

Continuous variables are presented as mean ± standard error, and categorical variables are presented as n (%)

^a^ Indicates statistical significance between the four groups

^b^ Adjusted for age and sex

^c^ Adjusted for age, sex, and total energy intake (continuous)

The proportion of participants who had moderate-intensity PA in the previous week was lowest in the sarcopenic obesity group (Table 4; *p* = 0.008). Likewise, the proportion of participants with vigorous-intensity PA was lowest in the sarcopenic obesity group (*p* < 0.001), which was significantly lower than in the sarcopenia group (*p* = 0.037). In terms of total time spent in PA after adjusting for age and sex, moderate-intensity exercise time (*p* < 0.001), vigorous-intensity exercise time (*p* = 0.004), and combined moderate- and vigorous-intensity exercise time (*p* < 0.001) were the lowest in the sarcopenic obesity group, followed by the central obesity group. Similarly, the proportion of participants who met the recommended PA level was lowest in the sarcopenic obesity group and highest in the healthy control group (*p* < 0.001).

**Table 4:** Physical activity of participants according to sarcopenia and central obesity status

	**Healthy control**	**Central obesity**	***p* value**	**Sarcopenia**	**Sarcopenic obesity**	***p* value**	***p* value**^**a**^
Moderate-intensity PA in the previous week			0.158			0.099	0.008
No	727 (66.5)	575 (70.4)		576 (73.1)	103 (81.1)		
Yes	439 (33.5)	283 (29.6)		238 (26.9)	29 (18.9)		
Vigorous-intensity PA in the previous week			0.002			0.037	< 0.001
No	929 (79.5)	725 (85.7)		699 (86.8)	122 (93.7)		
Yes	238 (20.5)	133 (14.3)		115 (13.2)	10 (6.3)		
Total minutes of moderate-intensity PA (min/wk)^b^	181.0 ± 19.4	131.6 ± 20.3	0.038	150.0 ± 21.3	52.2 ± 16.1	< 0.001	< 0.001
Total minutes of vigorous-intensity PA (min/wk)^b^	115.2 ± 16.5	75.2 ± 14.1	0.038	78.4 ± 11.7	45.0 ± 20.0	0.134	0.004
Total minutes of moderate-to-vigorous-intensity PA (min/wk)^b^^,c^	411.9 ± 43.3	281.9 ± 40.0	0.012	306.9 ± 36.8	142.2 ± 45.1	0.005	< 0.001
Level of moderate-to-vigorous-intensity PA			0.005			0.022	< 0.001
Inactive (No PA)	648 (58.7)	524 (64.7)		536 (68.7)	100 (79.2)		
Insufficient level of PA	106 (8.6)	92 (10.6)		56 (6.1)	13 (8.2)		
Recommended level of PA	413 (32.6)	242 (24.7)		222 (25.2)	19 (12.7)		

Abbreviation: *PA* physical activity

All data analyses conducted in the present study were based on weighted estimates with sample weight provided by KNHANES

Continuous variables are presented as mean ± standard error, and categorical variables are presented as n (%)

^a^ Indicates statistical significance between the four groups

^b^ Adjusted for age and sex

^c^ Includes sum of time spent in moderate-intensity and weighted vigorous-intensity (× 2) PA in the previous week

Table 5 summarizes the findings of the multivariate multinomial logistic regression analysis according to sarcopenia and central obesity status. After adjusting for potential confounders, increases in daily energy intake (per 100 kcal) were negatively associated with sarcopenia (OR: 0.956, 95% CI: 0.934–0.977) and sarcopenic obesity (OR: 0.964, 95% CI: 0.931–0.999). Participants with an energy intake below the EER had an increased likelihood of sarcopenia (OR: 1.616, 95% CI: 1.256–2.079) compared with those with an energy intake reaching or exceeding the EER. Protein intake yielded similar results, with the likelihood of sarcopenia decreasing with every 10 g increase in protein intake (OR: 0.933, 95% CI: 0.886–0.982). When protein intake did not meet EAR, the adjusted ORs of sarcopenia (OR: 1.329, 95% CI: 1.034–1.709) and sarcopenic obesity (OR: 1.608, 95% CI: 1.013–2.553) were significantly increased. However, this association was no longer significant when the model was further adjusted for total energy intake. Participants with inactive or insufficient PA levels had a higher likelihood of central obesity (OR: 1.513, 95% CI: 1.173–1.951) and sarcopenia (OR: 1.370, 95% CI: 1.052–1.784), particularly sarcopenic obesity (OR: 3.054, 95% CI: 1.612–5.784) when compared with those who met the recommended PA level. These associations persisted even after adjusting for total energy intake.

**Table 5:** Association of nutritional and physical activity factors with sarcopenia and central obesity status: results from multinomial logistic regression analysis

	**Model 1: AOR (95% CI)**^**a**^			**Model 2: AOR (95% CI)**^**b**^		
	**Central obesity**	**Sarcopenia**	**Sarcopenic obesity**	**Central obesity**	**Sarcopenia**	**Sarcopenic obesity**
Energy intake (per 100 kcal)	0.993 (0.973–1.014)	0.956 (0.934–0.977)***	0.964 (0.931–0.999)*			
Level of energy intake						
≥ EER	1.000	1.000	1.000			
< EER	1.122 (0.878–1.435)	1.616 (1.256–2.079)***	1.321 (0.815–2.140)			
Protein intake (per 10 g)	1.005 (0.965–1.048)	0.933 (0.886–0.982)**	0.930 (0.855–1.012)	1.043 (0.980–1.110)	1.026 (0.949–1.109)	0.984 (0.851–1.137)
Level of protein intake						
≥ EAR	1.000	1.000	1.000	1.000	1.000	1.000
< EAR	0.951 (0.742–1.219)	1.329 (1.034–1.709)*	1.608 (1.013–2.553)*	0.862 (0.649–1.144)	0.965 (0.718–1.295)	1.436 (0.784–2.630)
Level of moderate-to-vigorous-intensity PA						
Recommended PA Level	1.000	1.000	1.000	1.000	1.000	1.000
Insufficient or inactive PA levels	1.513 (1.173–1.951)**	1.370 (1.052–1.784)*	3.054 (1.612–5.784)***	1.505 (1.167–1.941)**	1.338 (1.024–1.750)*	2.996 (1.579–5.687)***

Abbreviation: *AOR* adjusted odds ratio, *CI* confidence interval, *EER* estimated energy requirement, *EAR* estimated average requirement, *PA* physical activity

All data analyses conducted in the present study were based on weighted estimates with sample weight provided by KNHANES

^a^ Adjusted for age, sex, marital status, education level, household income, smoking status, frequency of binge drinking, physical activity level, and diabetes

^b^ Adjusted for Model 1 + total energy intake (continuous)

^*^ *p* < 0.05

^**^ *p* < 0.01, and

^***^ *p* < 0.001

The association of the combination of energy intake, protein intake, and PA with sarcopenia and central obesity status is shown in Table 6. In terms of the combined effect of energy and protein intake, those who consumed both nutrients above the requirements had a 40% lower OR of sarcopenia (OR: 0.601, 95% CI: 0.444–0.814) than those who did not. However, this combined effect of energy and protein intake was not significantly associated with central obesity or sarcopenic obesity. In terms of the effect of combined energy intake and PA, the likelihood of central obesity and sarcopenic obesity decreased in participants with recommended PA levels regardless of energy intake, with ORs for central obesity (OR: 0.536, 95% CI: 0.391–0.736) or sarcopenic obesity (OR: 0.262, 95% CI: 0.114–0.602) being lowest in those with low energy intake and sufficient PA. On the other hand, the likelihood of sarcopenia decreased in participants with an energy intake reaching or exceeding the EER, regardless of whether their PA meets or does not meet the recommended level. In contrast to central obesity and sarcopenic obesity, the OR of sarcopenia (OR: 0.436, 95% CI: 0.290–0.655) was lowest in those who met energy and PA requirements.

**Table 6:** Association of combination of nutritional and physical activity factors with sarcopenia and central obesity status: results from multinomial logistic regression analysis

	**AOR (95% CI)**^**a**^		
	**Central obesity**	**Sarcopenia**	**Sarcopenic obesity**
Different energy and protein intake levels			
Energy intake < EER, protein intake < EAR	1.000	1.000	1.000
Energy intake < EER, protein intake ≥ EAR	1.134 (0.833–1.543)	1.038 (0.777–1.385)	0.586 (0.314–1.093)
Energy intake ≥ EER, protein intake < EAR	0.563 (0.263–1.206)	0.932 (0.523–1.661)	0.880 (0.253–3.054)
Energy intake ≥ EER, protein intake ≥ EAR	0.961 (0.723–1.278)	0.601 (0.444–0.814)**	0.635 (0.373–1.080)
Different energy intake and moderate-to-vigorous-intensity PA levels			
Energy intake < EER, insufficient or inactive PA levels	1.000	1.000	1.000
Energy intake < EER, recommended PA level	0.536 (0.391–0.736)***	0.744 (0.537–1.031)	0.262 (0.114–0.602)**
Energy intake ≥ EER, insufficient or inactive PA levels	0.774 (0.571–1.049)	0.634 (0.469–0.856)**	0.684 (0.398–1.176)
Energy intake ≥ EER, recommended PA level	0.678 (0.467–0.984)*	0.436 (0.290–0.655)***	0.316 (0.126–0.796)*

Abbreviation: *AOR* adjusted odds ratio, *CI* confidence interval, *EER* estimated energy requirement, *EAR* estimated average requirement, *PA* physical activity

All data analyses conducted in the present study were based on weighted estimates with sample weight provided by KNHANES

^a^ Adjusted for age, sex, marital status, educational level, household income, smoking, frequency of binge drinking, physical activity level, and diabetes

^*^ *p* < 0.05

^**^ *p* < 0.01 and

^***^ *p* < 0.00

## Discussion

This South Korean population-based study of the older adults aged ≥ 65 focused on body composition as defined by the presence of sarcopenia and central obesity. This approach differs significantly from the traditional conservative methods that examined obesity and sarcopenia as separate entities using only a single anthropometric index or did not distinguish sarcopenic obesity from sarcopenia, and it may be even more important and necessary in the older adults population. Only a few studies have focused on the combination of both of these conditions.

Sedentary or inactive lifestyles, as well as imbalances between energy intake and needs, which are well known to influence obesity, have been linked to the maintenance or increase of skeletal muscle mass [23, 24]. In this study, the combination of energy with protein and with PA was studied as the key to understanding the overriding determinants of central obesity, sarcopenia, and sarcopenic obesity. There were clear differences in energy and protein intake between the healthy control, central obesity, sarcopenia, and sarcopenic obesity groups. The energy intakes of the sarcopenia group, including sarcopenia and sarcopenia obesity, were lower than the Korean EER. In particular, those who did not meet the EER had a significantly higher likelihood of sarcopenia. However, those who did not meet the EAR for protein had a significantly higher likelihood of sarcopenia and sarcopenic obesity; these associations, however, were no longer statistically significant after adjusting for total energy intake.

Protein is one of the most effective nutrients for muscle synthesis, and adequate intake of protein, such as leucine-enriched amino acids, can promote skeletal muscle regeneration [25, 26]. However, in this study, energy-adjusted protein intake was not independently associated with sarcopenia or sarcopenic obesity. A previous Korean study, which was consistent with our findings, explained that it was due to the traditional Korean diet of the older adults, which is high in carbohydrates and low in animal protein [27, 28]. Furthermore, it is possible that people with low skeletal muscle mass are already on a deliberate diet, and the differences in protein intake between groups may not have been well detected because total energy intake was generally low (about 1,525 to 1,677 kcal) in all of these subjects. Energy, as well as protein, may play a role in the outcomes of a population with relatively low energy intakes, such as the Korean older adults in our study. Previous studies have demonstrated that increasing energy intake can help to maintain skeletal muscle mass and lower sarcopenia risk [23, 29]. Low energy intake was also linked to frailty in the older adults [30]. Several studies also found that the relationship between macronutrient intakes and frailty prevalence differed based on energy intakes. After adjusting for energy intake, the effect of protein intake on frailty was either eliminated [31] or reduced [30, 32, 33].

In this study, there was a difference in predicting the association of sarcopenia with combinations of energy intake and protein intake based on the accompanying abdominal obesity. We found that adherence to the recommendations for energy and protein intake was strongly associated with a lower sarcopenia prevalence. However, these findings did not apply to sarcopenic obesity, indicating that sarcopenic obesity may require a different treatment than isolated sarcopenia. Similarly, in the case of patients with sarcopenia who are malnourished, energy supplementation or increasing protein intake within the normal range of energy consumption may help to maintain skeletal muscle mass. Since skeletal muscle accounts for a substantial portion of the body’s total energy expenditure, providing enough calories may help to preserve skeletal muscle mass [23].

In terms of assessment of obesity, this study used the waist circumference which is widely accepted as an indicator of abdominal adiposity. Since the optimal cutoff value of body fat percentage has not yet been clearly specified for Korean adults, we were concerned that this cutoff value might overestimate obesity prevalence to some extent. The prevalence of obesity in this population, which was estimated using the criteria of the body fat percentage of the American Council on Exercise, was 32.1% in men and 66.5% in women. Moreover, the abdominal adiposity may be more associated with obesity-related diseases than whole body fat accumulation [34], and the waist circumference had a higher correlation with insulin resistance than percent whole body fat in the sample of healthy Korean adults [35].

Although the effects of combining energy and protein on sarcopenia or frailty have not been thoroughly investigated, it was observed that the total daily energy and protein intake were significantly lower in common among the older adults with low appendicular lean mass [36]. A community-based randomized controlled study was also conducted to investigate the effect of combining energy and protein intake. Protein-energy supplementation (an extra 400 kcal of energy, 25 g of protein, and 9.4 g of essential amino acids per day) given to the frail older adults slowed the progression of physical functional decline compared with controls who did not receive this supplement [37]. The Society for Sarcopenia, Cachexia, and Wasting Disease recommended adequate protein and energy intake as a key nutritional component for the prevention and management of sarcopenia [38].

In our study, physical inactivity was another factor that contributed significantly to both sarcopenia and central obesity. However, the combined effect of energy intake and PA produced different results for central obesity, sarcopenia, and sarcopenic obesity. Although the likelihood of central obesity, sarcopenia, and sarcopenic obesity was reduced in the group that met energy intake and PA recommendations, the positive combination of energy intake and PA appeared to greatly affect sarcopenia. Those with sufficient PA combined with a low energy intake had the most optimal association for central obesity or sarcopenic obesity. Furthermore, when only one of the two recommendations was met, PA at the recommended level was very important for both central obesity and sarcopenic obesity, whereas total energy intake to the EAR was more important for sarcopenia. Although data were not presented in the results, we also found that there were no specific sex differences in these patterns.

These findings imply that, while combining exercise and diet has a positive effect on both sarcopenia and obesity, the key determinants of sarcopenia and sarcopenic obesity may be different. Sarcopenia and obesity share several pathophysiological mechanisms [39], but the pathogenesis of sarcopenic obesity has yet to be investigated. In terms of natural history, sarcopenic obesity may follow a different path than sarcopenia [40]. However, few studies have compared biochemical status, lifestyle, and risk factors in people with sarcopenic obesity, isolated sarcopenia, or isolated obesity [41–43].

Previous studies, like this one, have revealed the importance of PA in sarcopenic obesity. A meta-analysis of randomized controlled trials demonstrated that exercise, particularly resistance exercise, is critical for improving body composition and physical performance in people with sarcopenic obesity [44]. A multi-continent study using nationally representative data from nine countries found that lower PA levels were significantly associated with both sarcopenia and sarcopenic obesity. Furthermore, people with sarcopenic obesity had lower PA levels than those with sarcopenia alone [41]. In Korean studies, all types of PA were found to be beneficial for both sarcopenia and sarcopenic obesity [42], and moderate-to-vigorous PA was found to be highly correlated with skeletal muscle index and hand grip strength [45].

The combined effects of exercise and nutrition on muscle strength, mass, and function have been investigated in interventional studies and systematic reviews [16, 46, 47]. The literature suggests that interventions based on providing an adequate energy supply and supplementing specific nutrients could be effective in either or both preventing and reversing sarcopenia and frailty, especially when combined with physical exercise [38, 47]. Furthermore, as in our study, the effects of combining energy-yielding nutrients with exercise were demonstrated in a study on Asians; post-exercise macronutrient supplementation (equivalent to 200 kcal) during home-based interval walking training enhanced increases in skeletal muscle mass and strength compared with exercise alone among middle-aged and older Japanese women [48]. Another study investigated the effects of caloric restriction-induced weight loss alone and in combination with moderate aerobic exercise on skeletal muscle mass; energy intake restriction for weight loss reduced skeletal muscle mass even more in the obese older adults, but adding moderate aerobic exercise to diet-induced weight loss reduced muscle mass loss [49]. These findings support the importance of exercise in the intervention of sarcopenic obesity.

There are several limitations to the present study. First, the AWGS 2019 algorithm for identifying and diagnosing older adults with sarcopenia requires measurements of both muscle quality and quantity, but the KNHANES dataset did not include hand grip strength data. Therefore, we could not consider muscle strength to define sarcopenia and adopted only low ASM as the diagnostic criterion. Second, due to the cross-sectional nature of the study design, the causal relationships among variables could not be determined. Third, we focused primarily on general recommendations for macronutrient intake and PA. However, more research is needed to understand the effects of micronutrients and specific components of exercise, such as exercise frequency, intensity, time, and type, in order to propose more effective strategies to counteract sarcopenia and obesity [21]. Fourth, as with any observational study, residual confounding by unmeasured or uncontrolled confounders existed and cannot be considered, such as age-related physiological factors, including changes in hormone levels, vascular changes, low-grade inflammation, and immunological factors that could contribute to the development of both sarcopenia and obesity [8]. Finally, we were unable to analyze the most recent KNHANES data due to the limited availability of data for bone density and body composition measured with DXA.

Despite these limitations, we investigated whether adherence to recommendations for energy intake, as well as protein intake or PA, was associated with a lower prevalence of obesity, sarcopenia, and sarcopenic obesity. Furthermore, the study samples included relatively healthy, non-institutionalized citizens (excluding severely ill patients and those already on diets) in a nationwide, population-based setting, rather than being limited to a specific community-based setting or sex group, which is an additional strength of our study. However, further studies are needed to longitudinally compare the combined effects of nutrition and exercise interventions in sarcopenia and sarcopenic obesity.

## Conclusion

In this study, the combined effect of energy intake, protein intake, and PA on sarcopenia and central obesity status in the Korean older adult population was investigated. Although adequate energy intake that meets requirements is more likely to be effective as a major treatment goal for sarcopenia, a combined strategy that considers both exercise and diet is required. Similarly, regular and active exercise at the recommended level should be considered an important treatment goal for sarcopenic obesity. Our findings provide insight into effective and optimal intervention strategies for the prevention and management of sarcopenia and sarcopenic obesity, both of which are major concerns in an aging society.

## Supplementary Information

**Additional file 1: Supplementary Figure 1. **Flow chart of selection of the study population. Abbreviation: KNHANES,Korea National Health and Nutrition Examination Survey; ASM, appendicular skeletal muscle mass.

## Data Availability

The data of the current study are available from the KDCA (https://knhanes.kdca.go.kr/knhanes/main.do) on reasonable request.

## Abbreviations

PA: Physical activity
OR: Odds ratio
CI: Confidence interval
KNHANES: Korea National Health and Nutrition Examination Survey
KDCA: Korea Disease Control and Prevention Agency
DXA: Dual-energy X-ray absorptiometry
ASM: Appendicular skeletal muscle mass
ASMI: Appendicular skeletal muscle mass index
EER: Estimated energy requirement
EAR: Estimated average requirement
KDRI: Korean Dietary Reference Intakes
HDL: High-density lipoprotein
BMI: Body mass index
