# Supplementary material for: The effect of combining nutrient intake and physical activity levels on central obesity, sarcopenia, and sarcopenic obesity: a population-based cross-sectional study in South Korea
Source: BMC Geriatr. 2023 Mar 3;23:119. doi: 10.1186/s12877-023-03748-x (PMC9985216; doi:10.1186/s12877-023-03748-x)
Supplement: Supplementary file 1 — Additional file 1: Supplementary Figure 1. Flow chart of selection of the study population. Abbreviation: KNHANES,Korea National Health and Nutrition Examination Survey; ASM, appendicular skeletal muscle mass. [file 12877_2023_3748_MOESM1_ESM.docx]

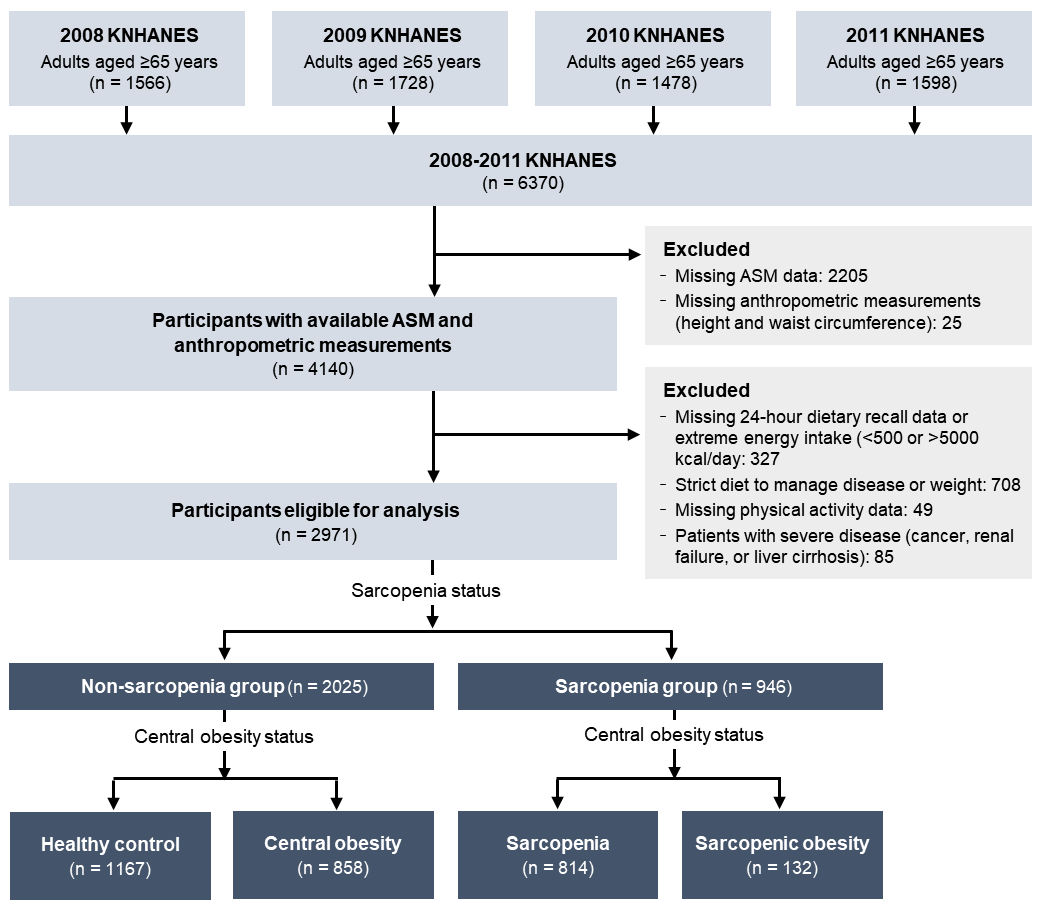


**Supplementary Figure 1**. Flow chart of selection of the study population.

Abbreviation: KNHANES, Korea National Health and Nutrition Examination Survey; ASM, appendicular skeletal muscle mass.
